# The Time Course of Language Production as Revealed by Pattern Classification of MEG Sensor Data

**DOI:** 10.1523/JNEUROSCI.1923-21.2022

**Published:** 2022-07-20

**Authors:** Francesca Carota, Jan-Mathijs Schoffelen, Robert Oostenveld, Peter Indefrey

**Affiliations:** ^1^Max Planck Institute for Psycholinguistics, 6525 XD Nijmegen, The Netherlands; ^2^Donders Institute for Cognitive Neuroscience, Radboud University, 6525 Nijmegen, The Netherlands; ^3^Institut für Sprache und Information at, Heinrich Heine University, Düsseldorf 40225, Germany; ^4^NatMEG, Karolinska Institutet, Stockholm 171 77, Sweden

**Keywords:** cascading models, conceptual preparation, language production, MEG, phonological/phonetic planning, sensor-level MVPA

## Abstract

Language production involves a complex set of computations, from conceptualization to articulation, which are thought to engage cascading neural events in the language network. However, recent neuromagnetic evidence suggests simultaneous meaning-to-speech mapping in picture naming tasks, as indexed by early parallel activation of frontotemporal regions to lexical semantic, phonological, and articulatory information. Here we investigate the time course of word production, asking to what extent such “earliness” is a distinctive property of the associated spatiotemporal dynamics. Using MEG, we recorded the neural signals of 34 human subjects (26 males) overtly naming 134 images from four semantic object categories (animals, foods, tools, clothes). Within each category, we covaried word length, as quantified by the number of syllables contained in a word, and phonological neighborhood density to target lexical and post-lexical phonological/phonetic processes. Multivariate pattern analyses searchlights in sensor space distinguished the stimulus-locked spatiotemporal responses to object categories early on, from 150 to 250 ms after picture onset, whereas word length was decoded in left frontotemporal sensors at 250-350 ms, followed by the latency of phonological neighborhood density (350-450 ms). Our results suggest a progression of neural activity from posterior to anterior language regions for the semantic and phonological/phonetic computations preparing overt speech, thus supporting serial cascading models of word production.

**SIGNIFICANCE STATEMENT** Current psycholinguistic models make divergent predictions on how a preverbal message is mapped onto articulatory output during the language planning. Serial models predict a cascading sequence of hierarchically organized neural computations from conceptualization to articulation. In contrast, parallel models posit early simultaneous activation of multiple conceptual, phonological, and articulatory information in the language system. Here we asked whether such earliness is a distinctive property of the neural dynamics of word production. The combination of the millisecond precision of MEG with multivariate pattern analyses revealed subsequent onset times for the neural events supporting semantic and phonological/phonetic operations, progressing from posterior occipitotemporal to frontal sensor areas. The findings bring new insights for refining current theories of language production.

## Introduction

The brief time interval preceding word production involves complex planning processes to transform a preverbal message into speech. How an intended meaning is rapidly mapped onto articulatory programs is, however, still disputed.

Two competing views of language production emerged from earlier studies. One account posits a hierarchically organized sequence of psycholinguistic operations with distinct latencies (Levelt, 1989, 1999; Levelt et al., 1999; Hagoort and Levelt, 2009). As estimated by relating chronometric data to metabolic studies of word production (Indefrey and Levelt, 2004), a target concept (e.g., APPLE) is accessed along with related concepts, such as PEAR, PEACH, FRUIT within the first 200 ms after picture onset in overt naming tasks. Once lexical information is selected, phonological codes are retrieved between 200 and 400 ms after picture onset in posterior middle/superior temporal cortex, and abstract segmental representations required for syllable planning (syllabification) are accessed at ∼400-600 ms, involving left inferior frontal activity. The target articulatory programs are retrieved ∼100 ms later, as reflected in premotor activity. Importantly, the abovementioned onset times reflect approximations only, and are influenced by characteristics of the to-be-pronounced words, such as word length, lexical frequency, and familiarity (Hanulovà et al., 2011; Indefrey, 2011), motivating a rescaling of the latencies on a word-by-word basis (Roelofs and Shitova, 2017).

Neurophysiological evidence (e.g., Salmelin et al., 1994; Levelt et al., 1998; Maess et al., 2002; Sörös et al., 2003; Vihla et al., 2006; Hultén et al., 2009; Liljeström et al., 2009; Laaksonen et al., 2012; for review, see Munding et al., 2016), and electrophysiological studies testing for reliability of language production tasks (e.g., Laganaro, 2017; Roos and Piai, 2020; Ala-Salomäki et al., 2021), confirmed the proposed cascade of functionally and temporally dissociable neural events.

In contrast, recent MEG results from picture naming suggested early (130-160 ms postpicture presentation), near-simultaneous occipital/temporal activations to visual complexity, and left inferior frontal and temporal activations to semantics and word form, respectively (Miozzo et al., 2015). Another MEG study found early modulation of frontotemporal activity by word frequency 160-240 ms postpicture presentation (Strijkers et al., 2017), and concurrent superior temporal activity reflecting differences in the phonetic/articulatory properties of a word's initial speech segment (either a labial or a coronal phoneme, as in monkey vs donkey, respectively). Furthermore, the place of articulation of the words' initial phoneme activated the motor regions controlling for lip and tongue movements, suggesting early articulatory planning.

In the light of these premises, here we asked to what extent the earliness of simultaneous brain responses to multiple psycholinguistic functions is an intrinsically distinctive property of the neural dynamics of word production.

To determine the spatiotemporal specificity of semantic and phonological planning, we combined the temporal precision of MEG with multivariate pattern analyses (MVPAs) in sensor space. Unlike univariate approaches, MVPA uses sensitive multivariate statistics to measure the covariation of signals across multiple neighboring channels at the single-trial level, and thus extracts information from distributed patterns rather than averaged amplitudes across selected channels. MVPA of MEG data also captures rich spatial information from the subtle multivariate magnetic field patterns of brain activity at sensor level, which result from small differences in orientation and angle of neighboring dipoles, and may overlap, but still be statistically separable (Stokes et al., 2015; Cichy and Pantazis, 2017). This allows for the differentiation of the subject-specific spatial topographies over subjects (Stokes et al., 2015).

We adopted an orthogonal design covarying word length and phonological neighborhood density, as targeting syllabification and phonetic encoding, across four semantic object categories (animals, foods, tools, and clothes) to test the following: (1) the serial/hierarchical prediction of early neural indexes of conceptual preparation, but later frontal activity of syllabification (350 ms) and phonetic encoding (450 ms after picture onset); and (2) the early/parallel prediction of early simultaneous activations to both conceptual and phonological/phonetic processes.

The present investigation aimed at probing the validity of these proposals to inform and refine current theories of language production.

## Materials and Methods

### Subjects

Thirty-four native Dutch speakers (26 male, mean age = 24 years, SD = 3.6 years) participated in the experiment after providing written informed consent. All subjects were right-handed, had normal or corrected-to-normal vision, and reported no history of neurologic, developmental, or language deficits. The study was approved by the ethical board CMO Arnhem/Nijmegen, under registration number CMO2014/288.

### Materials

Stimuli consisted of 134 images from four object categories, including animals, foods, tools, and clothes. We used colored realistic images from the picture database of Bank of Standardized Stimuli (Brodeur et al., 2014), and public domain images from the Internet (e.g., www.Freepng.ru).

Images were selected on the basis of a list of depictable target words that are most commonly used to name the corresponding objects in Dutch. The list of object words was generated by covarying the length of target words and phonological neighborhood density within each semantic category. Word length affects lexical (word form retrieval) and postlexical (syllabification, phonetic and articulatory encoding) processing stages in a straightforward quantitative way (Indefrey and Levelt, 2004; Papoutsi et al., 2009; Indefrey, 2011), that is, the more phonemes/syllables there are, the higher the load on the processes and the longer they take. Word form retrieval, for example, takes ∼20-25 ms per phoneme (Indefrey and Levelt, 2004; Indefrey, 2011). We included phonological neighborhood density because it affects lexical as well as postlexical phonological processes but via different mechanisms (Harley and Brown, 1998; Vitevitch, 2002; Dell and Gordon, 2003): whereas the facilitative effect of phonological neighbors on word form activation seems to be mediated by their shared phonological segments (Vitevitch, 2002), the facilitative effect of phonological neighborhood density at phonetic/articulatory processing stages seems to be because of its correlation with phonotactic probability (Vitevitch et al., 1999). Words with many neighbors tend to contain more common phonemes and phoneme sequences than words with fewer neighbors, making it easier to phonetically encode and articulate them. The effects of phonological neighborhood density on lexical retrieval and articulation have been shown to be independent and additive (Buz and Jaeger, 2016). A previous MEG study by Miozzo et al. (2015) has combined word length with phonological neighborhood density into a single predictor of neural activity related to word form access. For our investigation of the relative time courses of processes related to early stages of word production (conceptual preparation) and processes related to later phonological and phonetic stages, we therefore found it important to ensure comparability with the Miozzo et al. (2015) study by using the same variables to target phonological processing. At the same time, in view of the different nature of the effects of word length and phonological neighborhood density mentioned above, we reasoned that combining the two variables in one component might underestimate their specific effects, in particular, the effect of phonotactic probability captured by phonological neighborhood density but not by word length. We therefore decided to examine the two variables separately.

Word length was expressed by the number of syllables (65 short monosyllabic words, 66 long bisyllabic words, and 3 trisyllabic words). Phonological neighborhood density was expressed by the number of words that differ in one phone from the target word. This was calculated by counting all word entries in CELEX that differ in one phone symbol from the target word, after discarding stress and syllable markers from the phonological word representation in CELEX. Words were ranked according to such difference and subdivided into four groups (lower, low, high, higher) while keeping the group size as balanced as possible: 39 lower (0-4 phones), 29 low (5-9 phones), 35 high (10-19 phones), and 31 higher (20-39 phones). The psycholinguistic properties of the 134 stimulus items are summarized in Table 1. Mean and SD, median and range (the difference between largest and smallest value of the variable) are reported, as calculated across items and for mosyllabic and disyllabic words separately (Table 1).

**Table 1.** Psycholinguistic properties of the 134 stimulus items

Category	SUBTLEX word frequency		No. of syllables	No. of phonological neighbors	
	Mean (SD)	Median (range)	Mean (SD)	Mean (SD)	Median (range)
All words					
Animals	8 (10)	6.1 (49.9)	1.5 (0.6)	12.3 (10.1)	9 (45)
Clothes	9.8 (15.8)	4.2 (67.2)	1.5 (0.5)	11.7 (10)	8 (41)
Foods	5.7 (9.2)	2 (39.6)	1.5 (0.5)	12.2 (11)	9 (39)
Tools	9.4 (8.8)	6.8 (37.5)	1.6 (0.6)	11.9 (8.2)	11 (33)
All	8.3 (11.4)	4.8 (67.3)	1.5 (0.5)	12 (9.8)	9 (46)
Monosyllabic words					
Animals	11.1 (12.8)	7.5 (49.4)	19.1 (9.6)	18 (39)	
Clothes	17.6 (19.6)	11.6 (65.3)	17.9 (10.2)	17 (35)	
Foods	8.7 (12.3)	2.2 (39.5)	21.3 (9.0)	21 (29)	
Tools	13.7 (10.5)	10.8 (35.7)	16.9 (7.4)	15 (26)	
All	12.8 (14.4)	7.8 (67.1)	18.8 (9.1)	18 (39)	
Bisyllabic words					
Animals	5.4 (12.8)	2.9 (18.7)	6.1 (5.5)	4.0 (21)	
Clothes	2.5 (19.6)	1 (19.1)	5.8 (5.3)	3.5 (15)	
Foods	2.9 (12.3)	1.9 (10.1)	3.6 (2.8)	3 (9)	
Tools	5.1 (10.5)	5 (11.5)	8.0 (5.9)	7 (24)	
All	3.9 (14.4)	2 (19.2)	5.8 (5.1)	4 (26)	

Word length and phonological neighborhood density were negatively correlated with each other (*r*_(132)_ = −0.67, *p* < 0.001). Word frequency could not be fully matched across conditions. Word frequencies were obtained from SubtLex (Keuleers et al., 2010), which provides a standard measure of word frequency independent of corpus size: frequency per million words with a 4-digit precision. Word frequency was negatively correlated with word length (*r*_(132)_ = −0.37, *p* < 0.001) and positively correlated with phonological neighborhood density (*r*_(132)_ = 0.32, *p* < 0.001). Because this variable is known to affect all stages of the word production planning (Indefrey and Levelt, 2004; Hanulovà et al., 2011), we assessed the related statistical effect on our variables of interest, by conducting one-way repeated measure ANOVA with object category (animals, foods, tools, clothes), and word length (short, long), and Phonological Neighborhood density (low, high) as independent variables. There was no effect of word frequency on object category. As for the phonological variables, there was a main effect of word frequency on word length (*F*_(1,132)_ = 23.95, *p* < 0.001), with short words being more frequent (mean = 12.82) than long words (mean = 3.95). There was also a main effect of word frequency on phonological neighborhood density (*F*_(1,32)_= 11.69, *p* = 0.001), with low-density words being less frequent (mean = 5.07) than high-density words (mean = 11.53).

### Experimental design

Before starting the experiment, subjects received written and oral information about the picture naming procedure. Subjects were instructed to name aloud each object picture as fast and accurately as possible, and to speak clearly.

At the beginning of each experimental trial, a fixation cross was presented at the center of the screen for 500 ms, followed by a blank interval for 500 ms, and the object picture was subsequently presented for 350 ms. The 134 pictures were presented 3 times, once in each of three separate blocks, following a random order. The experiment lasted ∼30 min, including two self-paced breaks.

#### Behavioral recordings

Stimuli were presented using the Presentation software (Neurobehavioral Systems; www.neurobs.com). The pictures were displayed at the center of the screen at a size of 300 × 300 pixels (1920 × 1080 screen resolution and a refresh rate of 120 Hz, delay <1 ms with almost instantaneous presentation of the full screen), in a light gray background within a visual angle of 4 degrees. They were presented using a liquid crystal display video projector and back-projected onto the screen using two front-silvered mirrors.

Vocal responses were captured with a microphone and recorded at 44.1 kHz using the Audacity software (https://audacityteam.org/).

Vocal responses were recorded as wav files, and response latencies were determined offline using the Praat software (Boersma and Weenink, 2019).

#### MEG recordings

Subjects were seated in the MEG system in a magnetically shielded room. They were asked to sit comfortably but to keep their body and head still during the task, and to try to avoid blinking. They were instructed to look at the stimulus screen, located 40 cm in front of them, focusing on the center of the screen. The MEG signals were recorded using a high-density whole-head system (OMEGA 2000; CTF Systems), consisting of 275 axial gradiometer channels and 29 dedicated reference channels for environmental noise cancellation. The subject's head was registered to the MEG sensor array using three coils placed at three anatomic landmarks (nasion, and left and right ear canals). The head position was continuously monitored during the MEG recordings, and readjusted during breaks if it deviated >9 mm from the initial position (Stolk et al., 2013). Head movements did not exceed 1.25 cm between blocks. Pairs of Ag/AgCl electrodes were used to record the horizontal and vertical electro-oculograms, the electrocardiogram, and the surface EMG from the orbicularis oris muscle (electrodes placed above the upper lip and below the lower lip) (impedance was kept <15 kΩ for all electrodes). MEG, EMG, and electro-oculogram signals were analog low-pass filtered at 300 Hz, digitized at 1200 Hz, and saved to disk for offline processing.

### Data analysis

#### Behavioral data

Latencies of the subjects' verbal responses were calculated offline by subtracting the time of picture onset marked by a 10 ms auditory signal (inaudible to the participants) from the time of speech onset.

Praat software (http://www.praat.org) (Boersma and Weenink, 2019) was used to analyze the recorded audio signal and to semiautomatically identify the onset of beep and articulation. Automatic silence/nonsilence interval boundaries were obtained by using intensity (dB) thresholds, and the resulting onset boundaries were manually inspected and corrected where needed (most often at word-initial voiceless consonants or vowels); 83% of response trials were correctly named (identical to the target word). Incorrectly named trials (3%) and verbal disfluencies (stuttering, utterance repairs, and production of nonverbal sounds) (14%) were excluded from the analyses.

In order to assess and assure synchrony between the onsets of picture and of the auditory beep signal for subsequent MEG data analyses, the audio files were aligned with the picture onset triggers for the preprocessing of the MEG data, and with the audio channel in the MEG. For the alignment, the (very small) difference in clock speed of the computer for audio recording and the MEG acquisition computer was taken into account by estimating the delay between the presentation triggers and the delivery of the beep.

For behavioral data analysis, the effects of the variables of interest on naming latencies was assessed by conducting a one-way repeated-measures ANOVA on the averaged naming latencies of each subject with semantic category, word length, and phonological neighborhood size as independent variables.

#### MEG data preprocessing

Data were processed using MATLAB (version R2021a) and the FieldTrip toolbox (Oostenveld et al., 2011). Data were epoched into segments from −100 to 1000 ms relative to picture onset. Independent component analysis was used to remove ECG artifacts using the logistic infomax independent component analysis algorithm (Bell and Sejnowski, 1995), using the EEGLAB implementation (Delorme and Makeig, 2004) (http://eeglab.org). Before decomposing the MEG signal into components, data were bandpass filtered in the 1-30 Hz range and downsampled to 300 Hz. The topographies of the components were visually inspected, along with their time course for the first 40 trials, and the effect of removing the components that were identified as containing artifacts was checked. Samples contaminated by artifacts because of eye movements, muscular activity, and superconducting quantum interference device jumps were replaced by NaN (not a number) to allow excluding those samples from further analysis. For the sensor-level analysis, we calculated synthetic planar gradients using a nearest-neighbor interpolation (Bastiaansen and Knosche, 2000). Combining horizontal and vertical components of the estimated planar gradients allows for easier interpretation of the sensor-level results, as the maximal activity is typically located above the source (Hämäläinen et al., 1993).

#### Classification pattern analyses

Spatiotemporal MVPA was used to assess whether the experimentally manipulated stimulus features could be decoded from the MEG sensor signals. The stimulus features of interest were as follows: (1) object category; (2) word length as quantified by the number of syllables; and (3) phonological neighborhood density. The object category variable coded for the four categories of animals, tools, foods, and clothes; the word length variable coded for short (monosyllabic) and long (bisyllabic and trisyllabic) words; and the phonological neighborhood density variable was discretized into four classes of approximately equal size (smaller/small/large/larger). As such, decoding for the variables of interest constituted 4-way and binary classification tasks.

To control for low-level visual confounds, we decoded the object categories while accounting for low-level visual features of the object images in the estimation of the within-subject null-distribution of the category classification (see below). We took the following visual features into account: contrast, as measured by the intensity contrast between a pixel and its neighbors over the whole image (Corchs et al., 2016); visual complexity, quantified by edge density as the percentage of pixels that are edge pixels (Rosenholtz et al., 2007; Forsythe et al., 2008); and colorfulness, measured using distributional properties of pixel color values, as being strongly correlated with human judgments (*r* = .83) (Hasler and Suesstrunk, 2003). These variables were discretized into two classes of approximately equal size (low/high).

Additional *post hoc* analyses were conducted as a sanity check of the data to ascertain whether an uncontrolled variable, such as lexical frequency, yielded spatiotemporal patterns of classification that were similar to the patterns resulting from the main analysis, thus confounding the results. Word frequency was included because it is highly correlated with word length and neighborhood density.

We used a Gaussian naive Bayes classifier (Mitchell, 1997), as implemented in the MVPA-light toolbox (Treder, 2020). Despite its assumption that features are conditionally independent of each other given the class label (Bishop, 2006), this classifier has shown to be remarkably successful in a wide range of classification problems. To evaluate classification performance and to control for overfitting, repeated stratified five-fold cross-validation was used. The data were randomly split into five equal folds, ensuring the equal presence of classes in each fold (stratification). The model was trained on four folds and validated on the fifth fold. The process was repeated 5 times, such that each fold was used for validation. The entire cross-validation was furthermore repeated 5 times with new randomly assigned folds, to reduce bias that might be caused because of how data were randomly partitioned into folds, and the final averaged results are reported. To avoid classification bias because of class imbalance in the class labels, random undersampling was applied to training and test data, by discarding randomly selected samples from majority classes until each class was represented by an equal number of samples.

We quantified the decoding performance by means of classification accuracy, which is the fraction of correctly predicted class labels. The higher the classification accuracy, the better response patterns associated with the class labels can be determined.

We applied a spatiotemporal searchlight procedure and defined for each time point and channel a local searchlight, consisting of a 150 ms time window (45 data points at the downsampled sampling rate of 300 Hz), and the set of first order neighboring synthetic planar gradient channels. On average, each channel had 7.4 neighbors (SD = 1.1, range 4-10); thus, the classification at each channel-time point was based on 332 features (range 180-450). To assess whether classifier performance was above chance performance, we estimated the chance level empirically, using permutations at the single-subject level. We repeated the classification testing after shuffling the class labels, and recomputed classifier performance on the shuffled class labels to obtain a distribution under the null hypothesis of exchangeability of class labels (see, e.g., Cichy et al., 2014; Isik et al., 2014; Kaiser et al., 2016). The randomization of class labels for the number of syllables and the phonological neighborhood density classification was constrained to account for the fact that the object category was not fully orthogonal to the other features of interest. To this end, the randomization of class labels was performed for each object category separately.

We controlled for low-level visual confounds in the classification of semantic object categories, by constraining the within-subject randomization procedure (to obtain the subject-specific distribution for object classification under the null hypothesis) to binned collections of stimuli, where the bins were defined according to the visual features of the images. For statistical inference, we used nonparametric cluster-based permutation tests across space and time (Maris and Oostenveld, 2007), using 2000 permutations. The cluster-based permutation procedure uses the same spatial neighborhood structure that was used in the classification searchlight procedure, clustering the selected samples (sensors, time points) on the basis of spatial and temporal adjacency. The test statistic used was a group-level *T* statistic against the empirical chance level using the subject-specific *Z*-standardized decoding accuracy scores. These *Z* scores for each subject were obtained by subtracting the mean accuracy obtained from 100 randomizations from the observed accuracy, and dividing by the SD across randomizations.

## Results

### Behavioral results

Table 2 reports the average RT and naming accuracy per object category, as quantified based on subjects' actual responses during the MEG experiment. One-way repeated-measures ANOVA on the average naming latencies of each subject with object category, word length, and phonological neighborhood size as independent variables revealed a main effect of object category (*F*_(1,32)_ = 12.6, *p* < 0.001), with the RTs being slower for clothes (see Table 2).

**Table 2.** Averaged RTs and naming accuracy values per object category

	RT (ms)		Accuracy (% correct)	
Category	Mean	SD	Mean	SD
Animals	831	90	84.3	12.9
Clothes	891	100	67.3	12.2
Foods	841	100	84.7	10.2
Tools	834	80	88.2	12.3

The analyses also revealed a main effect of word length on the RT (*F*_(1,32)_ = 7.34, *p* = 0. 011), monosyllabic words (mean = 838 ms, SD = 98) showing a shorter RT than bisyllabic words (mean = 855 ms, SD = 092). Neighborhood density also showed a main effect (*F*_(1,32)_ = 7.09, *p* = 0.012), as words with a high number of phonological neighbors showed shorter RTs (mean = 838 ms, SD = 86) than those with a low number of phonological neighbors (mean = 854 ms, SD = 95).

Furthermore, we inspected whether object categories interacted with word frequency. As expected, high-frequency words (mean = 821 s, SD = 85) were named faster (*F*_(1,32)_ = 50.43, *p* < 0.001) than low-frequency words (mean = 877 s, SD = 102). Importantly, though, there was no interaction of word frequency with object category, suggesting that the semantic effects were not linked to word frequency.

### Results from MVPA searchlight analysis on MEG data

**Figure 1.**
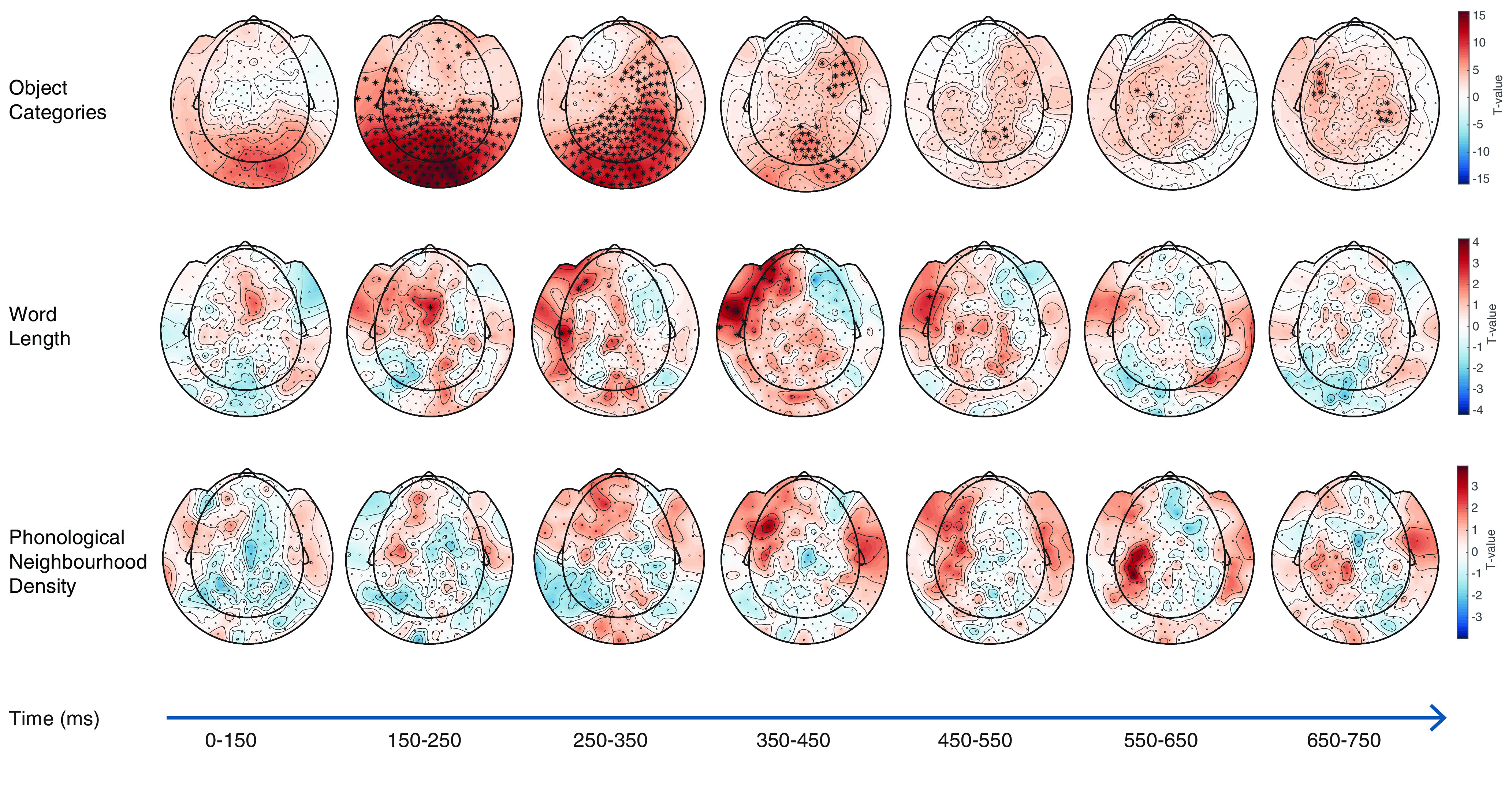
Topographic maps displaying the effect of decoding accuracy (expressed as a *T* statistic) for each key variable: (1) object categories (top), (2) word length (middle), and (3) phonological neighborhood density (bottom). Data points exceeding the nominal cluster threshold at *p* < 0.05 (uncorrected) are marked with an asterisk. Object category information was decoded early on, starting in the time window from 150 to 250 ms, across a distributed set of bilateral occipitoparietal, and left temporal sensors. From the subsequent time window (250-350 ms), word length was decoded in frontotemporal sensor area, while phonological neighborhood density was decoded in left frontal sensors from 350 to 450 ms. For phonological neighborhood density, no data points exceeded the *a priori* cluster threshold for multiple comparisons correction.

**Figure 2.**
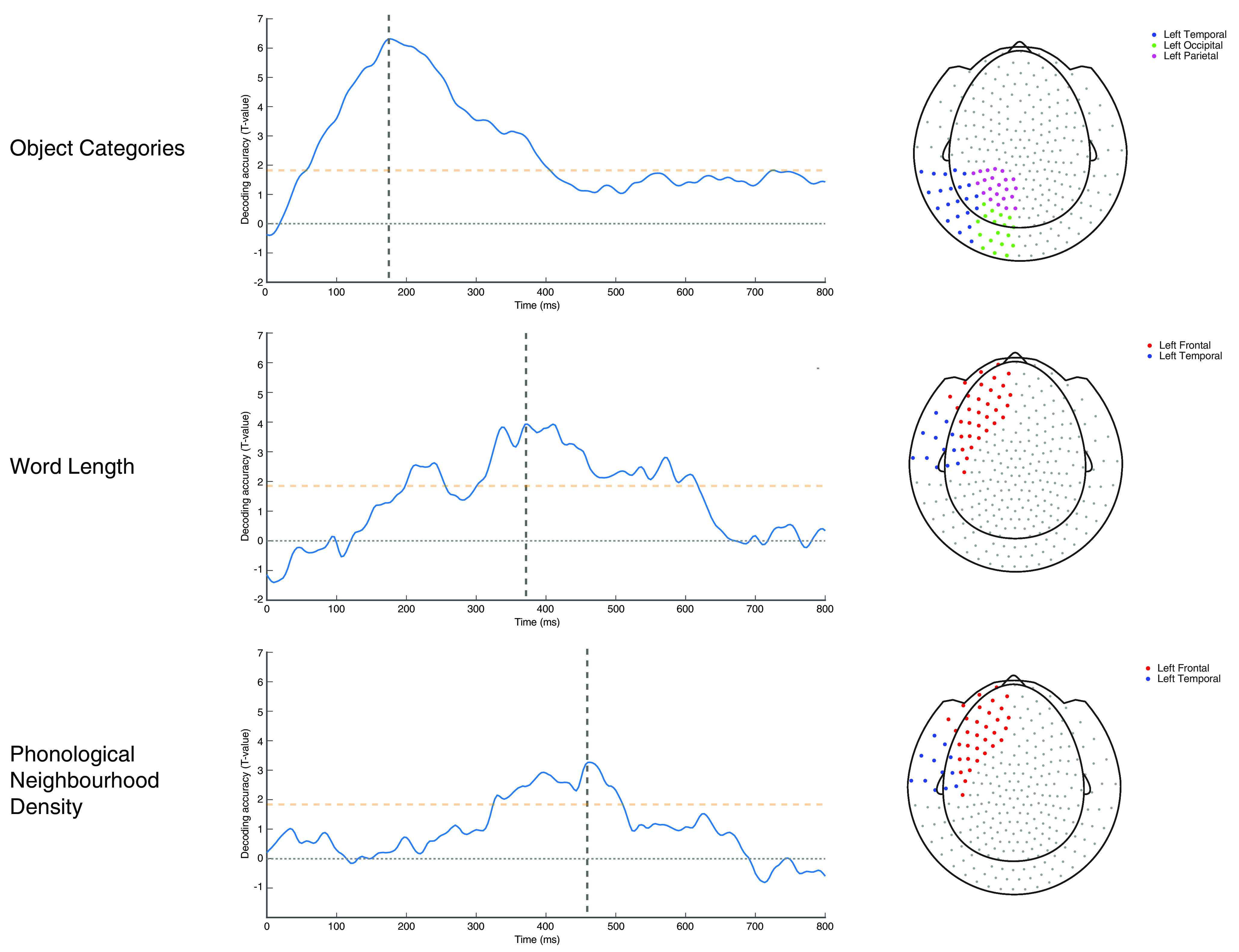
Time courses of decoding accuracy for the key study variables (left panels), peaking at subsequent times (as marked by vertical bars). Horizontal dashed bars in yellow represent the *a priori* cluster threshold for uncorrected *p* < 0.05 for decoding accuracy. Data are extracted from left occipitoparietal and posterior temporal sensors areas for object category (top), from left frontal and adjacent anterior temporal sensors for both word length (middle) and phonological neighborhood density (bottom), as shown in the topographic views on the right. The sensor selection was based on *a priori* hypotheses on the emergence of semantic information in posterior occipitoparietal and temporal regions, and of phonological processes in left frontal regions, as proposed by the Indefrey and Levelt (2004) model.

Results from the spatiotemporal searchlight analyses further revealed specific neural dynamics associated with the key semantic and phonological variables of the present study. Decoding outcomes yielded above chance raw accuracy values of 37% for object category, 56% for word length, and 28% for phonological neighborhood density. Although raw accuracy values were not high, they were above chance. Figures 1 and 2, respectively, display the topographic distributions and the time course of decoding accuracy for each contrast of interest. We found that the four object categories were significantly distinguished early on, starting in the time window 150-250 ms, in bilateral occipital, temporoparietal, central, and left frontal sensor areas, as shown in Figure 1 (top).

In contrast, above chance decoding accuracy for word length was first observed in a central sensor area starting in the time window 150-250 ms, and later, starting in the time window 250-350 ms, in left temporal and frontal sensors (Fig. 1, middle). Starting in the subsequent time window (350-450 ms), decoding accuracy for phonological neighborhood density was significantly above chance over a more focused subset of left frontal sensors (Fig. 1, bottom).

The corresponding time courses of decoding accuracy from selected left occipitoparietal and posterior temporal sensors for object categories, and from left frontal and adjacent anterior temporal sensors for word length and phonological neighborhood density are displayed in Figure 2.

**Figure 3.**
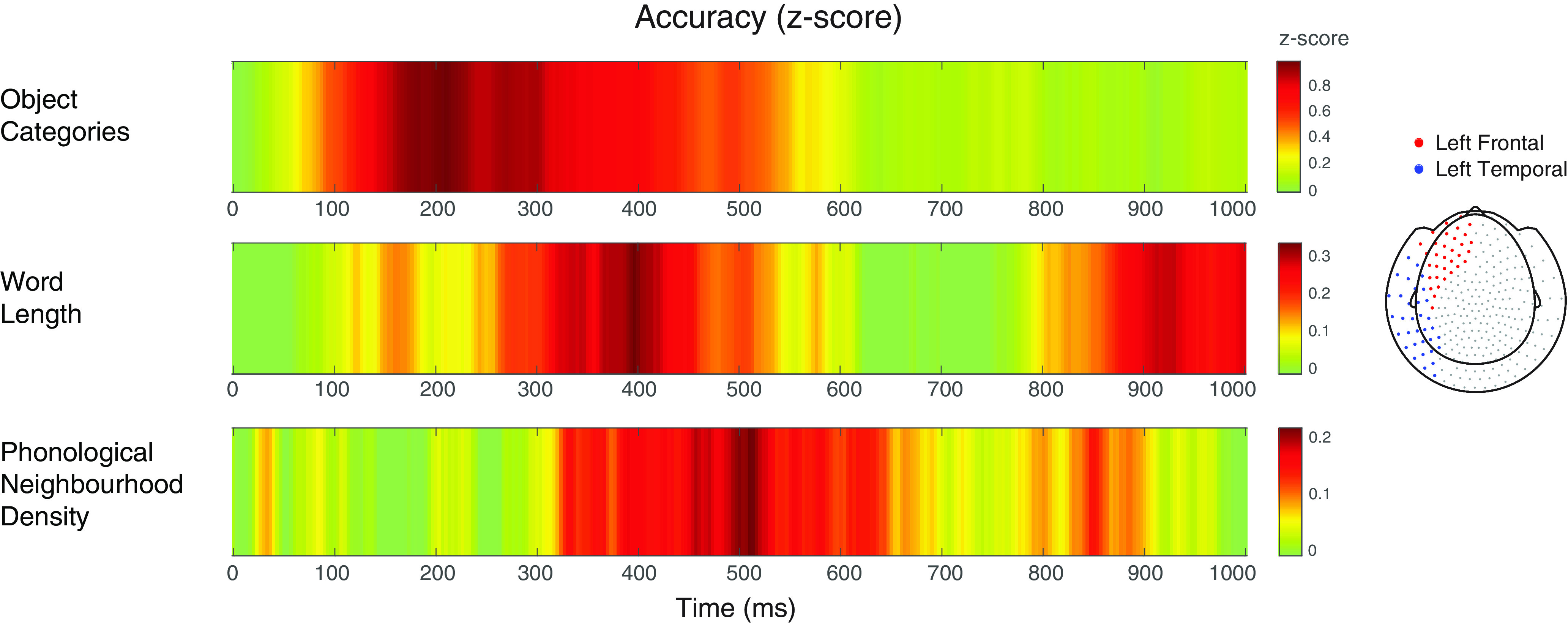
Overview of the searchlight results visualised as time course of decoding accuracy across left frontal and temporal sensors (as displayed in the topographic view on the right of the figure) for the key study variables: object category (top), word length (middle), and phonological neighborhood density (bottom). For each contrast of interest, the color bar represents the *Z*-standardized decoding accuracy values, from zero values (in green), to increasingly positive *Z* scores (up to dark red).

The searchlight results for the key study variables are summarized as time courses of decoding accuracy (*Z*-standardized values) across left frontal and temporal sensors in Figure 3.

**Figure 4.**
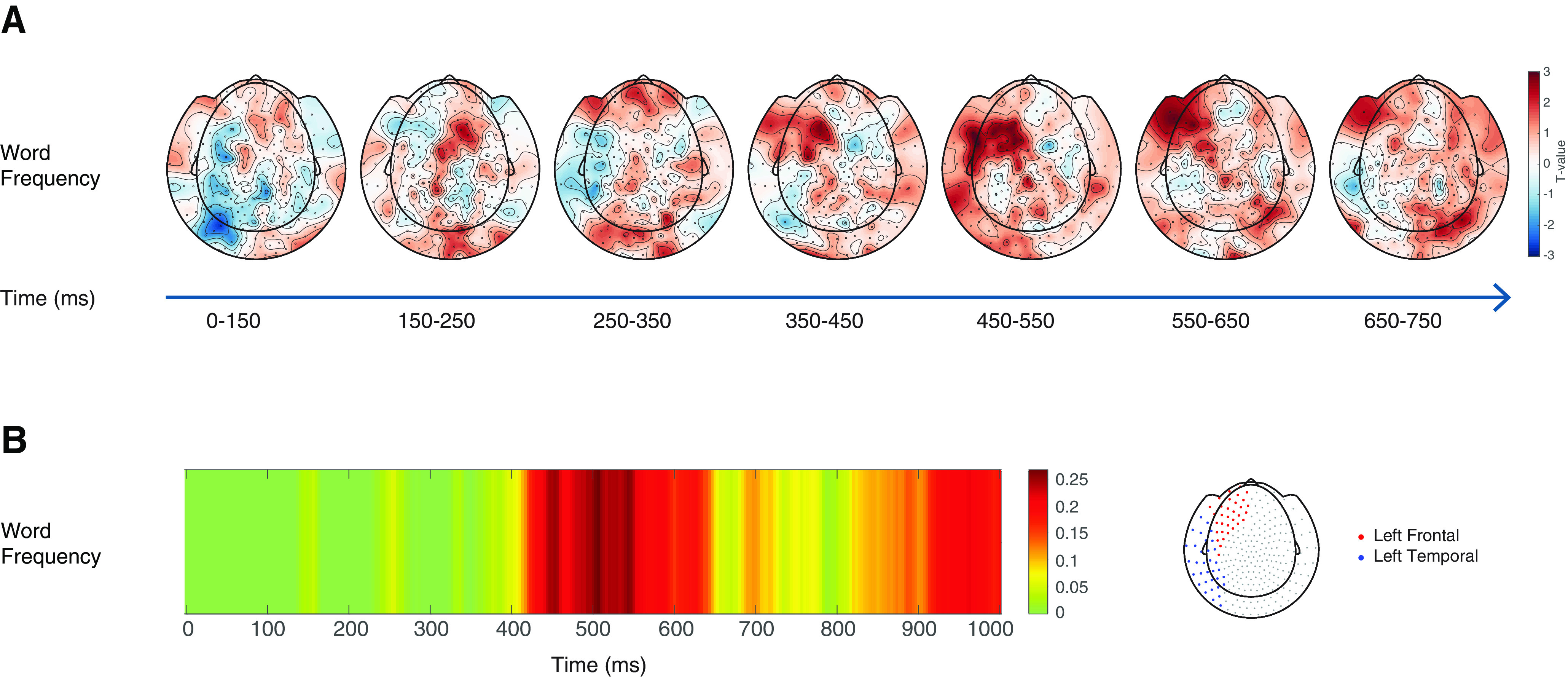
***A***, Topographic maps displaying the effect of decoding accuracy (*T* statistic) for word frequency. For each contrast of interest, the color bar represents the *Z*-standardized decoding accuracy values from zero values (in green), to positive *Z* scores (up to dark red). Note the early activity arising from central sensors for the word frequency condition, in a time window that was comparable to the effects in central sensors found for word length. ***B***, Overview of the searchlight results displayed as time course of decoding accuracy across left frontal and temporal sensors (as displayed in the topographic view on the right of the figure) for word frequency.

For the control variable of word frequency, decoding accuracy was above chance in central sensors in an early time window (Fig. 4*A*), which was comparable to the spatiotemporal effects observed for word length. Above chance accuracy for word frequency across left frontal and temporal sensors was, however, only seen from a later time window (450-550 ms), as displayed in Figure 4*B*, suggesting differences in the underlying processes.

## Discussion

Using MVPAs of the MEG data, we investigated the time course of neural activity underlying semantic and phonological access in object naming. Conceptual preparation was cued by categorical distinction of the stimulus objects, while syllabification and phonetic encoding were, respectively, linked to the manipulation of word length and phonological neighborhood density. Our method revealed discriminability of object categories in occipitoparietal and temporal sensors early on (150-250 ms). Word length was decoded from the subsequent time window (250-350 ms) in left frontotemporal sensors, followed by phonological neighborhood density (350-450 ms).

Regarding the competing serial/hierarchical and early/parallel hypotheses, based on the target variables of the present study, our results speak in favor of a cascade of neural events with sequential onset times for the conceptual, phonological, and phonetic processes that enable overt object naming.

The observed early differentiation of object categories during conceptual preparation is consistent with established knowledge that brain activity in the ventral visual pathway rapidly evolves from the visual perception and recognition of object images in posterior occipital and parietal cortex to the perceptual feature conjunctions enabling the representation of conceptual meaning in inferior temporal and fusiform cortex, as identified by previous MEG work (Clarke et al., 2013). In relation to the sketched continuum from perception to conceptualization in the visual regions, the initial activity sparking from occipital sensors to parietal and posterior temporal sensors was expected to reflect visual perceptual processes from which categorical object representations emerge during the early stages of object meaning identification (Carlson et al., 2014). Recent studies have shown that perceptual similarity predicts object categories more reliably than semantic category at early stages of visual object comprehension (Proklova et al., 2019; Contini et al., 2020). The perceptual properties of the visually presented objects can then obviously confound the decoded activity in language tasks (Vindiola and Wolmetz, 2011; Simanova et al., 2014, 2015). As our data were controlled for basic visual features during classification of object categories, we found no decoding effects in the semantic object category condition in the first time window (0-150 ms). We interpret the results from the second time window onward as being predominantly driven by semantic processes linked to object meaning identification and categorization, consistent with the prediction from the Indefrey and Levelt (2004) model regarding conceptual preparation. The present study could thus effectively decode semantic object category information about objects at sensor level independently of the visual modality (Haxby et al., 2001; Cox and Savoy, 2003; Kamitani and Tong, 2005; Reddy and Kanwisher, 2007) with which an object is presented, in line with previous MVPA studies from whole-head high-density MEG signals (Simanova et al., 2010; Chan et al., 2011; van den Nieuwenhuijzen et al., 2016). The replicability of decoding of object categories in the present study also corroborates earlier evidence that patterns in sensor space carry information about object categories with latencies ∼150-250 ms after picture presentation (for review, see Contini et al., 2017).

Our results determined the relative earliness of conceptually driven preparatory activity with respect to the later latencies of phonological processes during word production planning, confirming MVPA pattern-classification of MEG data as being a reliable tool to investigate the time course of the neural dynamics in language tasks, such as overt naming.

A second result of importance of our present work concerns the time course of neural activity associated with phonological information, as word length was decoded from left frontotemporal sensors, from 250 to 350 ms, while phonological neighborhood density from left frontal and temporoparietal sensors was decoded 160 ms later, from the time window 350-450 ms. In line with the Levelt and Indefrey's model, these phonological operations started immediately after the onset time expected for phonological code retrieval. Also, their spatial distribution confirms consolidated evidence for a role of frontal regions in phonological processes (Fiez et al., 1999; Fiebach et al., 2002; Hagoort, 2005; Carreiras et al., 2006; Hauk et al., 2006; Graves et al., 2007; Papoutsi et al., 2009; Wilson et al., 2010), and a role of temporal regions in mapping auditory and motor representations (Hickok et al., 2003; Hickok and Poeppel, 2007), and in phonological/phonetic encoding (de Zubicaray et al., 2002; Gorno-Tempini et al., 2004, 2008; Bles and Jansma, 2008; Henry and Gorno-Tempini, 2010; Wilson et al., 2010), both in production and in perception (e.g., Obleser et al., 2003; Indefrey and Levelt, 2004; Hickok and Poeppel, 2007; Indefrey, 2011; DeWitt and Rauschecker, 2013; Mesgarani et al., 2014).

Interestingly, later left temporoparietal activity was specific to neighborhood density (at ∼550 ms after picture onset), and may be because of self-monitoring processes supported by the corresponding brain regions (e.g., McGuire et al., 1996; Hirano et al., 1997; Shergill et al., 2002; Tourville et al., 2008). Earlier neuroimaging studies have shown a specific modulation of activity in superior temporal cortex by words with high neighborhood density (Okada and Hickok, 2006). Words with a phonological neighbor may reduce neural activation in a network of superior temporal, inferior parietal, inferior frontal, and precentral regions, reflecting a facilitatory effect in lexical selection, phonological and articulatory planning, because of the overlap between the phonological representation of the target word and of its competitor neighbor (Peramunage et al., 2011; see also Harley and Brown, 1998).

Overall, spatiotemporal properties of the neural activity in our data diverge from parallel models suggesting early concurrent activity for conceptual and phonological information because of the ignition of cells in multiple regions for multiple functions (Miozzo et al., 2015; Strijkers and Costa, 2016; Strijkers et al., 2017). Rather, the present results provide evidence that conceptual preparation and phonological/phonetic encoding trigger activity in the language systems at subsequent time intervals. Our findings therefore support hierarchical models with temporal separation of onsets at the semantic and phonological planning stages.

In the present results, transient activity in central sensors became manifest in the topographic distribution of neuromagnetic activity for word length 150-250 ms. Central sensors notably pick up activity in medial frontal regions, such as (pre-) supplementary motor area (SMA), and/or cingulate cortex, which earlier studies have reported for similar tasks. For instance, the SMA plays diverse roles in lexical selection, phonological sequencing, articulatory (Dronkers, 1996; Jurgens, 2009; Tremblay and Gracco, 2010), and self-monitoring (e.g., Möller et al., 2007), along an anterior-to-posterior anatomic gradient (Alario et al., 2006). However, the existing literature provides inconsistent evidence for the time course of activity and functions of this region in overt naming. In a previous metabolic study, Wilson et al. (2010) found activity modulation of SMA and premotor regions as a function of word length, and speculatively proposed it to reflect access to articulatory scores. Another picture naming study using MEG identified a pattern of connectivity linking medial frontal regions, supramarginal gyrus, and posterior middle temporal cortex at ∼300 ms after picture presentation (Liljeström et al., 2015), and suggested a role of the SMA in early control processes for articulatory planning. At similar latencies (300 ms after picture onset), electrophysiological work reported SMA activity linked to vocal onset as an index of motor preparation in speech (Riès et al., 2013).

With respect to these earlier studies, the effect in central sensors in our data occurred even earlier, when previous studies found early phonological (Miozzo et al., 2015) and articulatory activations (Strijkers et al., 2017). This issue deserves further investigation. Still, in the context of our present study, we reasoned that, if the central sensor activity was linked to purely phonological or phonetic processes, we would observe a similar early effect also for the other phonological variable we used here, phonological neighborhood density, which was however not the case. Since we carefully manipulated our stimulus set for our dimensions of interest, but we could not control for lexical frequency, for which *post hoc* analyses indicated early effects in central sensors, we interpret these early dynamics as reflecting differences in intercorrelated lexical properties, such as word length and lexical frequency (short high-frequency words vs long low-frequency words), a variable known to influence behavioral performance in picture naming, and related neural activity exactly in middle frontal regions and cingulate cortex in both young and old adults (Gertel et al., 2020).

In conclusion, using MVPA searchlights in sensor space, the present MEG study demonstrated that MEG sensor patterns carry information about both semantic object categories and phonological processes during word production planning. The results delineated a spatiotemporal dynamics of semantic access, syllabification, and phonetic encoding characterized by modulation of activity in posterior occipitoparietal and temporal sensors by the different conceptual categories early on, and in left frontal and temporal sensor areas by the two phonological variables at later time windows. The time course of the neural events linked to these core word planning operations largely confirms a progression of neural activity from posterior to anterior regions of the language network along the axis from conceptualization to phonological/phonetic operations. Our results support serial cascading models of word production.

Our approach combining MEG and single-trial MVPA spatiotemporal searchlights offers a promising method for investigating the spatiotemporal properties of language planning to evaluate and refine current models of language production.
